# Computational Fluid Dynamics as a Digital Tool for Enhancing Safety Uptake in Advanced Manufacturing Environments Within a Safe-by-Design Strategy

**DOI:** 10.3390/ma18020262

**Published:** 2025-01-09

**Authors:** Dionysia Maria Voultsou, Stratos Saliakas, Spyridon Damilos, Elias P. Koumoulos

**Affiliations:** Innovation in Research & Engineering Solutions (IRES), 1000 Brussels, Belgium; dvoultsou@innovation-res.eu (D.M.V.); esaliakas@innovation-res.eu (S.S.); sdamilos@innovation-res.eu (S.D.)

**Keywords:** computational fluid dynamics, simulation, turbulent flow, particle tracing, safety, manufacturing, three-dimensional printing

## Abstract

In modern manufacturing environments, pollution management is critical as exposure to harmful substances can cause serious health issues. This study presents a two-stage computational fluid dynamic (CFD) model to estimate the distribution of pollutants in indoor production spaces. In the first stage, the Reynolds-averaged Navier–Stokes (RANS) method was used to simulate airflow and temperature. In the second stage, the Lagrangian method was applied for particle tracing. The model was applied to a theoretical acrylonitrile butadiene styrene (ABS) filament 3D printing process to evaluate the factors affecting the distribution of ultrafine particles (30 nm). Key parameters such as ventilation system effects, the presence of cooling fans and the print bed, and nozzle temperatures were considered. The results show that the highest flow velocities (1.97 × 10^−6^ m/s to 3.38 m/s) occur near the ventilation system’s inlet and outlet, accompanied by regions of high turbulent kinetic energy (0.66 m^2^/s^2^). These conditions promote dynamic airflow, facilitating particulate removal by reducing stagnant zones prone to pollutant buildup. The effect of cooling fans and thermal sources was investigated, showing limited contribution on particle removal. These findings emphasize the importance of digital twins for better worker safety and air quality in 3D printing environments.

## 1. Introduction

With the introduction of manufacturing technologies such as 3D printing, the challenges in protecting health and safety have increased. Three-dimensional printing releases ultrafine particles (UFP, <100 nm in diameter), which mainly result from the use of thermoplastic materials [1]. The UFP released during printing can reach rates of billions of particles per minute [2]. In contrast with the more commonly studied microscale particles, UFPs deposit predominantly in the alveolar and tracheobronchial regions of the respiratory tract where due to their high reactivity attributed to the high relative surface area, they can induce pulmonary inflammation and the generation of reactive oxygen species (ROS) [3]. UFPs deposited on the alveoli can be absorbed in the bloodstream and through translocation affect the function of several organs [4]. Additionally, particles smaller than 20 nm have the potential to even penetrate non-damaged skin, leading to systemic absorption [5], while particles under 10 nm can be absorbed by the olfactory nerve, increasing the risk of neurodegenerative disorders [6].

Predicting and reducing exposure to airborne pollutants is a complex issue due to the dynamics of airflow and pollutant mixing in enclosed spaces. In this context, computational fluid dynamics (CFD) is a valuable tool as it provides an accurate analysis of flow distribution and pollutant concentrations. CFD enables the understanding of the aerodynamic behavior of pollutants, improving the ability to predict and control the atmosphere in production facilities. These technologies allow engineers and those responsible for health and safety to design more efficient ventilation and filtration systems, reducing the risk of worker exposure to harmful pollutants and improving air quality in workplaces [7,8].

Since the 1970s, computational fluid dynamics was first used in built spaces with the aim of predicting air movement and flow velocities. Since then, its application has expanded to various fields, from industrial facilities to hospitals and homes [9]. In industrial plants, the intensive use of mechanical equipment poses significant health risks and indoor environmental concerns. Flynn et al. used computational fluid dynamics (CFD) to simulate contaminant concentrations near the breathing zone during spray painting in a ventilated booth. They developed a model to calculate airflow velocity and an algorithm to convert particle trajectories into pollutant concentration data. The study found that their CFD model accurately represented pollutant concentration changes based on ventilation conditions, confirming CFD’s effectiveness in predicting pollutant exposure [10]. Wang et al. used a CFD model with the discrete phase model (DPM) to analyze particle removal from air. They applied the renormalization group (RNG) k-ε model to simulate airflow while the Lagrangian method was used to track the particle trajectories. By testing different air supplies and exhaust speeds, they observed that combining advection with exhaust improved ventilation efficiency by 20–40% and that optimizing these speeds could achieve up to 70% energy savings while maintaining particle removal efficiency [11]. Wang et al. studied particle concentration transport by simulating the dispersion of oil mist in a processing plant under mixed ventilation. They used the Eulerian and Lagrangian methods to calculate oil mist concentration. Both methods provided acceptable results for the ventilation systems. However, the Lagrangian method increased computation time by over 53% and required 400 times more computational resources than the Eulerian method, with a 10% increase in error in cases of increased vorticity. Both methods gave reliable results for oil mist particles with an aerodynamic diameter of 0.5 μm [12]. In addition, CFD has been successfully applied in healthcare settings for infection prevention. The study by D’Alicandro et al. showed that laminar airflow systems are highly effective in reducing the concentration of ultrafine particles in operating rooms, helping to reduce the risk of infections [13]. In residences, Li et al. applied artificial intelligence techniques, such as a back-propagation neural network combined with an adaptive multi-objective optimizer (AMOPSO), to address CO_2_ and PM2.5 concentrations [14].

The integration of CFD into Industry 4.0 technologies offers extensive possibilities and applications. In the context of Industry 4.0, the role of CFD has evolved through the adoption of digital twins (Ds), artificial intelligence (AI) and mixed reality (MR) technologies. According to Diederich et al., integrating AI with CFD enhances simulation accuracy and reduces computational cost, leading to simplified models that predict fluid dynamics with high accuracy [15]. In addition, research by Boettcher et al. [16] and Mourtzis et al. [17] highlights how MR technologies create immersive environments for engineers, facilitating interactions with complex CFD simulations. These developments have practical applications in all industries. This cutting-edge approach combines CFD with optimization techniques, unlocking new possibilities for effective pollutant control.

The purpose of this study is to develop a computational model capable of predicting the effects of airflow and process parameters (such as heat sources and mechanisms affecting airflow in the room) on ultrafine particle distribution, offering a cost-effective solution for the manufacturing of advanced pilot lines. While previous studies have explored airflow and particle distribution separately, there is a significant gap in integrated models that account for both airflow dynamics and process parameters to predict particle behavior in complex manufacturing environments. This knowledge is useful in guiding abatement strategies such as ventilation system speed adjustments, the use of separation zones or walls, the adjustment of process parameters, and the application of personal protective measures to reduce operator exposure to particles, especially when their presence in the space is inevitable.

## 2. Mathematical Modeling

### 2.1. Numerical Model

**Figure 1:**
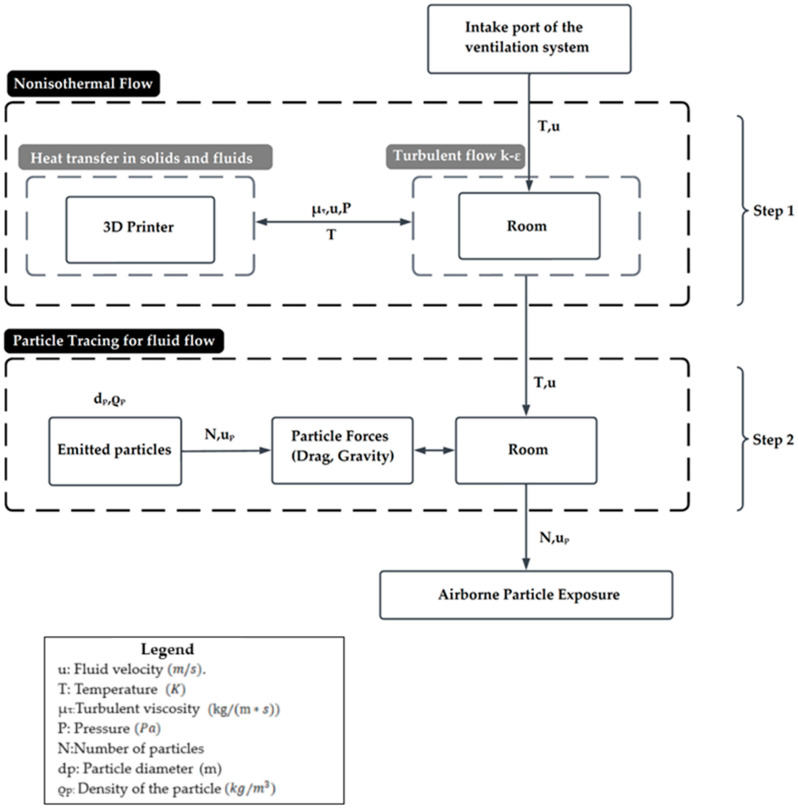
Diagram of the interconnected modules and variables for the two steps of the FEM model developed in COMSOL Multiphysics.

The analyses of airflow, heat transfer, and particle tracking were performed in the COMSOL Multiphysics software tool (v6.2, COMSOL Inc., Burlington, MA, USA). The COMSOL Multiphysics software is a powerful simulation tool that utilizes the finite element method (FEM) to solve complex physics-based problems across multiple domains. The computational analysis was divided into two steps and is based on the one-way coupling approach of COMSOL Multiphysics. The one-way coupling approach simplifies simulations by modeling airflow and particle dispersion independently. Here, airflow affects particle movement, but particles do not influence airflow. It assumes particles are passive tracers, making it effective for analyzing pollutant dispersion without feedback effects. Figure 1 shows the diagram of the interconnected computational steps. In the first step, the non-isothermal flow part was solved for the air velocity profile and temperature distribution within the room. This was achieved by solving the mass, momentum, and energy conservation equations, while not including particle emission activity. The temperature distribution and airflow velocity profiles calculated in this step were later used to simulate flow and particle behavior. In the second step, the particle tracking module was solved, aiming to calculate the trajectory of the particles emitted during the 3D printing process and their respective velocity magnitudes. The particle trajectories inside the room were calculated based on the airflow velocity profile obtained in the first step, thus ensuring that the interaction of the airflow with the particles is directly related to the temperature distribution and flow characteristics.

A steady flow field fluid study was used to obtain the velocity within the room. The physics chosen was a single-phase turbulent k-ε flow formulation. The direct segregated solver setup was used in the simulation. The solution was considered converged when the residual values fell below 10^−6^ (relative and absolute tolerances). A transient Newtonian formulation incorporating the Stokes law of attraction was used to model the behavior of the particles. For the analysis, the direct configuration of a fully coupled solver was used. The simulation was performed on a computer with a 12th Gen Intel(R) Core (TM) i9-12900KF processor at 3.20 GHz, with 16 cores and 64 GB of memory, meeting the computational requirements of the project. The total simulation time was 13 h and 25 min, of which 10 h were dedicated to the steady-state solver (non-isothermal flow) and 3 h and 25 min to the transient solver (particle tracing for fluid flow).

### 2.2. Governing Equations

To accurately simulate the internal airflow in this study, one of the three main CFD methods for analyzing turbulent flows in closed environments was used: the Reynolds-averaged Navier–Stokes (RANS) method. In particular, the k-ε model was applied, which is one of the most widespread ways of simulating turbulent flow. Additionally, the k-ε model is relatively easy to combine with Lagrangian particle tracing methods because the turbulence variables immediately give estimates of the amplitude of the velocity perturbations due to turbulent eddies (proportional to k/ε) and the average eddy lifetime (proportional to $\sqrt{k}$). For stationary, weakly compressive flow, the equations solved are as follows:

Continuity equation

(1) $$𝛻\left(\rho u\right)=0$$

where ρ is the fluid density $(kg/m^{3}),$ and u is the fluid velocity (m/s).

Momentum equation

(2) $$\left(u𝛻\right)u=𝛻\left[-pI+Κ\right]+F$$

(3) $$Κ=\left(\mu +\mu_{\tau }\right)+\left(𝛻u+{\left(𝛻u\right)}^{T}\right)-\frac{2}{3}\left(\mu +\mu_{\tau }\right)\left(𝛻u\right)I-\frac{2}{3}\rho kI$$

where p is the pressure (Pa); F is the external force $\left(N\right);$ μ is the fluid dynamic viscosity $\left(Pa/s\right),$ and I represents the identity matrix.

Turbulent viscosity $\mu_{\tau }(kg/\left(m*s\right))$ is given by the following formula:

(4) $$\mu_{\tau }=\rho C_{\mu }\frac{k^{2}}{\varepsilon }$$

where $C_{\mu }$ is a model constant.

The transport equation for k, the turbulent kinetic energy ($m^{2}/s^{2}),$ is as follows:

(5) $$\rho \left(u𝛻\right)k=𝛻\left[\left(\mu +\frac{\mu_{\tau }}{\sigma_{\kappa }}\right)𝛻k\right]+P_{k}-\rho_{\varepsilon }$$

where $\sigma_{k}$ is a model constant, and $P_{k}$ is the production term.

(6) $$P_{k}=\mu_{\tau }\left(𝛻\;u:{\left(𝛻u+(𝛻u\right)}^{T})-\frac{2}{3}{\left(𝛻\;u\right)}^{2}\right)-\frac{2}{3}\rho k𝛻u$$

The transport equation for ε turbulent dissipation rate ($m^{2}/s^{3})$ is as follows:

(7) $$\rho (u𝛻)\varepsilon =𝛻\left[\left(\mu +\frac{\mu_{\tau }}{\sigma_{\varepsilon }}\right)𝛻\varepsilon \right]+C_{\varepsilon 1}\frac{\varepsilon }{k}P_{\kappa }-C_{\varepsilon 2}\rho \frac{\varepsilon^{2}}{k}$$

where $C_{\varepsilon 1}$,$C_{\varepsilon 2}$, and $\sigma_{\varepsilon }$ are a model’s constants.

The values of the constants are as follows: $C_{\varepsilon 1}=1.44$, $C_{\varepsilon 2}=1.92$, $\sigma_{k}=1$, and $\sigma_{\varepsilon }=1.3$.

Energy Equation

(8) $$\rho C_{p}u𝛻T+𝛻q=Q$$

(9) q=−κ𝛻Τ

where $C_{p}$ is the specific heat capacity (J/(kg∗K)); Τ is the temperature (K); q is the heat flux vector ($W/m^{2})$, and Q is the rate of heat generation per unit volume $\left(W/m^{3}\right)$.

The present study describes particle detection through a Lagrangian approach, solving Newton’s equations of motion for particle trajectories in a time domain. The forces acting on the particles are determined at each time step by external fields. The process is repeated for each time step, with updates to the particle position, taking into account effects such as interactions with geometry boundaries. The equation of motion for a particle is based on Newton’s second law, according to which the mass of the particle times the acceleration equals the vector sum of all the forces acting on it. In this case, only two forces are considered: gravitational force (or net weight) and aerodynamics. Therefore, Newton’s second law can be expressed as follows:

(10) $$\frac{d}{dt}\left(m_{p}v\right)=F_{t}=F_{g}+F_{D}$$

where $F_{t}$ is the total force acting on the particle; $F_{g}$ is the gravitational force, and $F_{D}$ is the drag force.

The net gravitational force is equal to the weight of the particle minus the buoyant force on the particle, where the buoyant force is equal to the weight of the air displaced by the particle. For a spherical particle, the following equation is used:

(11) $$F_{g}=m_{p}g-m_{air}g$$

where $m_{p}$ is the particle mass (kg); $m_{air}$ is the fluid mass (kg), and g is the acceleration due to gravity.

Assuming the particles do not change in size (thus, $d_{p},$ και, and $m_{p}$ are stable), the mass of a sphere is expressed as follows:

(12) $$m_{p}=\frac{\pi }{6}{\;\;\rho }_{p}d_{p}^{3}$$

(13) $$F_{g}=\frac{\pi }{6}{\;\rho }_{p}d_{p}^{3}g-\frac{\pi }{6}{\rho d}_{p}^{3}g=\frac{\pi }{6}d_{p}^{3}(\rho_{p}-\rho )g$$

where $\rho_{p}$ is the density of the particle $(kg/m^{3});$ ρ is the fluid density $(kg/m^{3})$; $m_{p}$ is the particle mass (kg), and $d_{p}$ is the particle diameter.

Consider a particle moving with some arbitrary velocity v through the air at a position where the air is moving with some other arbitrary velocity u along an air stream. As the particle moves relative to the air, the air produces an aerodynamic drag force on the particle. For particles of diameter $d_{p}$, the drag force can be written as follows:

(14) $$F_{D}=-C_{d}\frac{\rho }{2}\frac{\pi d_{p}^{2}}{4}\left(u-v\right)$$

where $C_{d}$ is the drag coefficient.

The Schiller–Naumann drag law applies at a moderate level relative to

(15) $$C_{d}=\frac{24}{Re}(1+0.158\;{Re}^{2/3}).$$

This correlation is commonly used in indoor particle detection models [18,19,20].

The Reynolds number is defined in terms of the relative velocity and diameter of the particles:

(16) $$Re=\frac{\rho d_{p}\left(u-v\right)}{\mu }$$

For a spherical particle of diameter $d_{p}$, the net gravitational force is given by Equation (13), and the drag force is given by Equation (14). Substituting these expressions into Equation (10) yields the equation of motion of a single spherical particle in air:

(17) $$\frac{dv}{dt}\frac{\pi }{6}{\;\rho }_{p}d_{p}^{3}=\frac{\pi }{6}d_{p}^{3}(\rho_{p}-\rho )g-C_{d}\frac{\rho }{2}\frac{\pi d_{p}^{2}}{4}\left(u-v\right)$$

(18) $$\frac{d^{2}q}{dt^{2}}=\frac{\rho_{p}-\rho }{\rho_{p}}g+\frac{1}{\tau_{p}}(u-v)$$

where $\tau_{p}$ has units of time and is usually called the Lagrangian time scale. When the drag law is set to the Schiller–Naumann configuration, the particle velocity response time is redefined as follows:

(19) $$\tau_{p}=\frac{4\rho_{p}d_{p}^{2}}{3\mu C_{d}Re}$$

### 2.3. Computational Domain and Mesh

**Figure 2:**
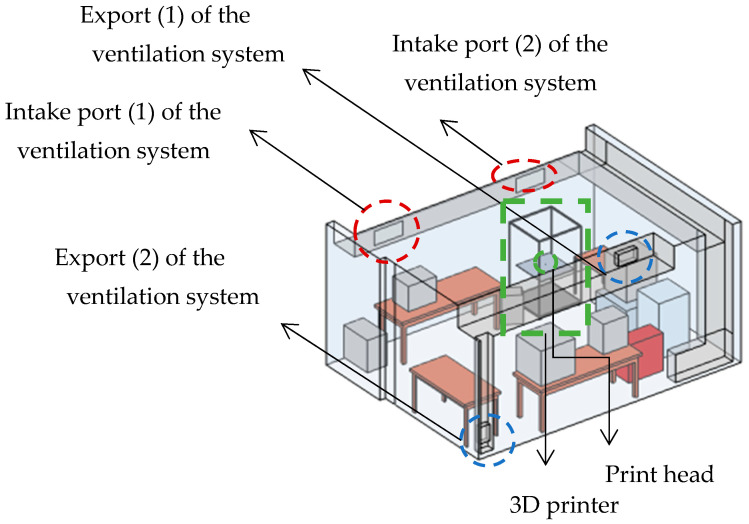
Schematic of the test room. The 3D production facility, where the inlets and outlets of the ventilation system are highlighted, as well as the studied 3D printer and the print head.

Figure 2 shows the computer-aided design (CAD) model of the 3D printing production room, with dimensions of 4.1 × 5.5 × 2.8 m, where the 3D printer and other necessary infrastructures such as a ventilation system, machineries, workbenches, and additional 3D printers. The Modix BIG-120Z 3D (Modix Modular Technologies Ltd., Central District, Israel) printer used in this work is specially designed for large-scale printing and supports a variety of filaments.

Air enters the room through the ventilation system intake ports, as shown in Figure 2, and exits through the exports of the same system, as shown in Figure 2. In the simulation, a set of boundary conditions was applied to accurately represent the physical environment of a 3D printing production space. Given the importance of effectively managing the factors that affect the distribution and removal of particles in space, variations in the speed of the ventilation system were meticulously incorporated, as well as in the operating temperatures of the 3D printer and the operation of the cooling fans. The inlet conditions for the ventilation system air ducts were set at velocities of $u_{1}=1.69\;m/s$, $u_{2}=0.64\;m/s$, $u_{1}=0.39\;m/s$, and $u_{2}=0.15\;m/s$, respectively, with a constant inlet temperature of 20 °C. Table S1 (Supplementary Information) shows the boundary conditions and parameters used in the simulations of the CFD model. To simulate the release of ultrafine particles during 3D printing, acrylonitrile butadiene styrene (ABS), one of the most commonly used materials in 3D printing, was chosen due to its strength and thermal stability [21]. As an amorphous thermoplastic, ABS has a relatively low melting point (210–270 °C), and for optimal material flow, the nozzle temperature is typically set at 250 °C, while the print surface temperature is set to 80 °C to enhance the grip of the ABS. Despite its high mechanical demands, particle emission rates for ABS were estimated to exceed $10^{11}$ particles per minute [22]. Based on previous studies on UFP emissions during ABS 3D printing, the particle size was set at 30 nm [23,24]. To avoid excessive computational demands that would arise from modeling over a billion particles, a smaller number, comprising 6010 particles, was chosen to be released over a 1-minute interval. The total simulation time was set at 600 s. During the first 60 s, 6010 particles are released, with a time step of 0.1 s. This release process occupies 10% of the total simulated time, while the remaining 90% (540 s) is dedicated to monitoring the performance of the ventilation system in removing particles from the air.

The core approach for computational simulations and verifying a numerical analysis involves identifying and quantifying errors in the computational solution. A critical aspect of verification testing is the systematic refinement of grid size. Consequently, a mesh refinement study was conducted as part of the verification process. To ensure the stability of the convergence of the numerical calculations, a grid dependency analysis was performed using the grid convergence index (GCI) [25]. The GCI, which was first proposed by Roache in 1994, is based on the Richardson expansion principle [26]. Ever since its introduction, GCI has been widely used in various numerical simulations such as construction [25,27], ventilation and pollutant dispersion [28,29,30], aerodynamics [31], and turbine design [32,33]. Despite the increasing number of elements or different mesh styles studied in the mesh refinement, a critical variable must be selected for the refinement simulation [34]. Therefore, depending on the numerical simulations, different crucial variables have been selected in various studies, such as temperature [27], air velocity [35], noise level [36], etc. Analyzing the techniques for indoor air quality evaluation, Tan et al. [37] reviewed the methods for airflow and particle dispersion, stating the importance and applicability of GCI methodology to minimize numerical errors, while highlighting the significant impact of appropriate model selection as well as boundary and wall conditions on the accuracy of the results.

In our work, grid dependency studies were conducted based on the GCI to assess the numerical accuracy obtained from the grid analysis. To determine the discretization accuracy error, the model was combined with three grids of different sizes to cover a range from 6 thousand to 5 million cells. Initially, in the CFD model, three basic grid sizes (Table 1) of different resolutions (coarse, medium, and fine) were selected.

The mesh improvement factor was determined as follows [38,39]:

(20) $$r=\frac{N_{coarse}}{N_{fine}}$$

where $N_{fine}$ represents the finer mesh. If $N_{1}<N_{2}<N_{3}$, where $N_{1}$ is the number of elements of coarse mesh; $N_{2}$ is the number of elements of medium mesh, and $N_{3}$ is the number of elements of fine mesh, the following dependencies were proposed to determine the mesh refinement factors:

(21) $$r_{21}=\frac{N_{2}}{N_{1}}$$

(22) $$r_{21}=\frac{N_{3}}{N_{2}}$$

The convergence order p was determined by following the dependence in [39]:

(23) $$p=\frac{\left|\ln\left|\frac{e_{32}}{e_{21}}\right|+\ln\left(\frac{r_{21}^{p}-1\;sgn\left(\frac{e_{32}}{e_{21}}\right)}{r_{32}^{p}-1\;sgn\left(\frac{e_{32}}{e_{21}}\right)}\right)\right|}{ln(r_{21})}$$

where

(24) $$e_{32}=c_{3}-c_{2}$$

(25) $$e_{21}=c_{2}-c_{1}$$

While c denotes the value of the crucial variable for the specific numerical simulation of the solution obtained for each base grid size. In further calculations, the velocity at the outlets of the ventilation systems ($C_{1},{\;C}_{2},\;and\;C_{3}$) was chosen as the critical variable.

The approximate relative error was estimated as follows:

(26) $$e_{\alpha }^{21}=\left|\frac{c_{1}-c_{2}}{c_{1}}\right|$$

(27) $$e_{\alpha }^{32}=\left|\frac{c_{2}-c_{3}}{c_{2}}\right|$$

The GCI is defined by the following equation:

(28) $${GCI}_{21}=\frac{F_{s\;}e_{\alpha }^{21}}{\left({r_{21}}^{p}-1\right)}$$

where Fs is a safety factor. For three or more meshes in CFD simulations, the recommended value is equal to 1.25 and represents a 95% confidence level in the calculated error bound [39]. All the values of the above-defined quantities are listed in Table 2.

According to the GCI calculation procedure, after selecting three base grid sizes with varying resolutions to validate the numerical computation results, it was determined that the fine and coarse GCI values were 24% and 8%, respectively. The reduction between these GCI values indicated the most dependable results. A grid size of 5,584,269 elements was identified as the optimal choice for simulations. The mesh composition includes 4,799,502 tetrahedra, 81,351 pyramids, 714,053 prisms, 216,668 triangles, 380 quadrilaterals, 18,046 edge elements, and 429 vertex elements. The element sizes range from a maximum of 0.285 (m) to a minimum of $9.78\times 10^{-4}$ (m), providing fine details where needed. The average element quality is 0.6718, with a minimum quality of 0.0272.

## 3. Results and Discussion

### 3.1. Airflow, Heat, and Particle Dispersion Simulations

**Figure 3:**
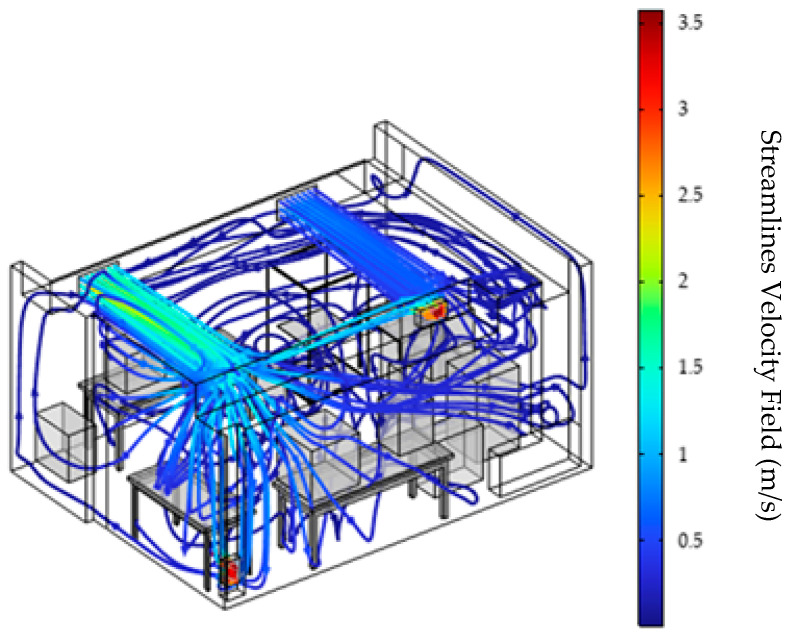
Visualization of streamlines in the 3D printing room with air velocities at the ventilation system inlets: u_1_ = 1.69 m/s and u_2_ = 0.64 m/s.

Figure 3 illustrates the streamlines from the numerical CFD simulation, providing an overview of the aerodynamic field throughout a space. In the simulation, a set of boundary conditions was applied to accurately represent the physical environment of a 3D printing production space. Given the importance of effectively managing the factors that affect the distribution and removal of particles in the space, variations in the speed of the ventilation system were meticulously incorporated, as well as in the operating temperatures of the 3D printer and the operation of the cooling fans. The inlet conditions for the ventilation system air ducts were set at velocities of $u_{1}=1.69\;m/s$ and $u_{2}=0.64\;m/s$, respectively, with a constant inlet temperature of 20 °C. Flow velocities range from $1.97\times 10^{-6}\;m/s$ to  3.38 m/s, with the highest values observed near the vent’s inlet and outlet. This observation is in line with the literature findings which support that these areas are characterized by high flow zones that enhance air circulation, contributing to a more uniform velocity distribution by optimizing ventilation and minimizing stagnation zones [40,41,42]. High levels of turbulent kinetic energy are concentrated around the inlets and outlets, enhancing air dispersion (Figure S2, Supplementary Information). This intensive flow helps improve air quality and overall ventilation efficiency by promoting even distribution and reducing stagnant areas where air circulation is limited or non-existent, causing pollutant build-up. Stagnant areas are usually created by the insufficient or incorrect distribution of air in a space, such as poor air duct placement or obstructions. Eliminating stagnant areas improves air quality and the overall efficiency of the ventilation system [43]. In the center of the 3D printing production room, air velocities are lower, averaging around 0.14 m/s, indicating a smoother flow and gentler air movement. In addition, the presence of vortices, which enhance the instability of the flow and contribute to the creation of eddies, is observed. These eddies, also known as dead zones, can cause pollutants to accumulate, adversely affecting air quality [44,45].

**Figure 4:**
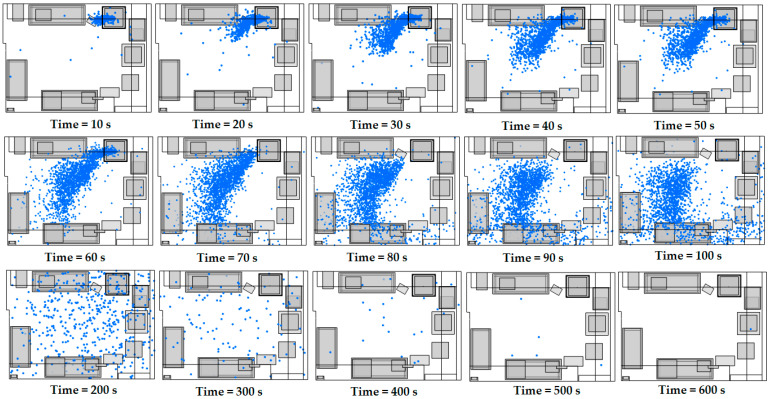
A top-down view of particle distribution in the 3D printing facility between 10 and 600 s, with the following air velocities at the ventilation system inlets: u_1_ = 1.69 m/s and u_2_ = 0.64 m/s.

In Figure 4, we observe the distribution of the ultrafine particles in the floor plan of the room. In the initial phase, from 0 to 60 s, a sharp increase in the total number of particles is observed, which is associated with the intense emission of particles from the source. In this phase, the particles are mainly concentrated around the source, with limited dispersion in the rest of the room. However, soon the dispersion of particles begins to expand rapidly and covers larger areas of space. From 60 to 300 s, the particle distribution becomes more uniform throughout the room, moving away from the source area. The cessation of the release of new particles from the source contributes to their spread throughout the room, specifically towards the outlets of the ventilation system. In these areas, high levels of turbulent kinetic energy are observed, enhancing air circulation and facilitating the effective removal of particles. At the final time points (400, 500, and 600 s), the particle concentration decreased drastically, with very few particles remaining in the room. This development suggests that ventilation mechanisms and flow direction are instrumental in removing particles, reducing their concentration even in areas with milder air circulation.

The literature presents a wealth of studies examining the effect of heat sources on indoor particle distribution and transport, highlighting the critical importance of temperature in air quality control and pollutant reduction. These investigations mainly focus on how heat can enhance physical convection and affect airflow, facilitating the removal of particles [46,47]. However, in the field of 3D printing, the research emphasis is mainly on local temperature effects related to the quality and integrity of printed parts. Temperature is mainly considered as a factor affecting adhesion and material properties during printing and not so much as a factor affecting the wider distribution and removal of particles in a space [48,49]. However, the temperature gradients created by heat sources facilitate heat transfer through conduction and initiate air convection currents. As the nozzle heats up and rises due to buoyancy effects, it creates convection currents. These air currents can carry extremely fine particles away from the immediate vicinity of the print area, dispersing them throughout the larger print space. There is a gap in the literature regarding the effect of temperature during the 3D printing process, especially in the dispersion and removal of released particles. The model we developed in this paper allowed us to systematically investigate the effect of temperature on the dispersion of particles in the room. The results show a small increase in temperature locally at the level of the print bed and in the areas near the print head, but this increase does not significantly affect the overall dispersion and movement of particles in the room (Figures S3 and S4, Supplementary Information). It appears that the heat sources affect the dispersion of the particles mainly in the first few seconds after their release, as a slightly increased dispersion of the particles was recorded immediately after their emission. However, in the following seconds of the cleaning process from the ventilation system, the heat sources do not affect the distribution and dispersion of the particles at all. Although small, this contribution demonstrates the positive effect of heat in reducing the concentration of particles, supporting the maintenance of air quality in 3D printing spaces. These findings highlight the need for further investigation into the role of temperature in printing conditions

### 3.2. Sensitivity Analysis of Air Flow Velocity Parameter

**Figure 5:**
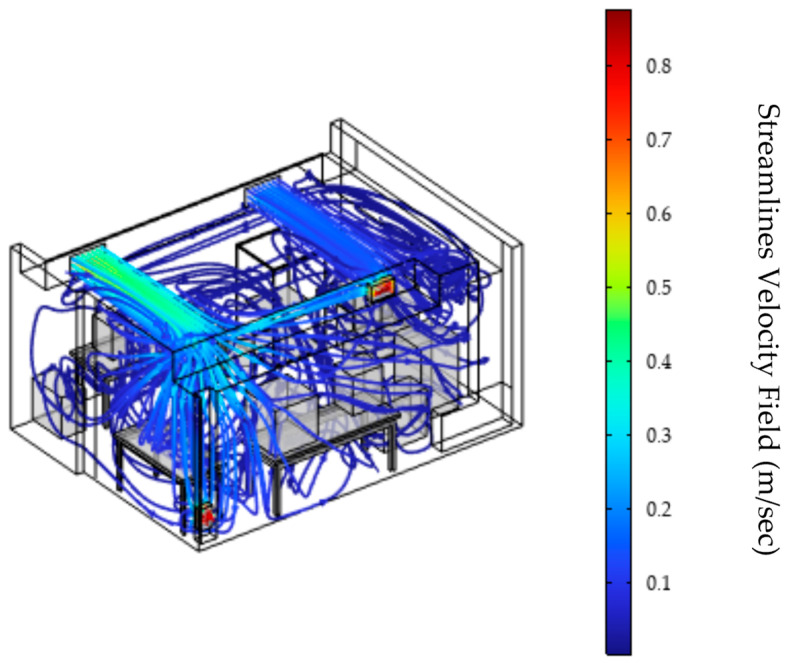
Visualization of streamlines in the 3D printing room with air velocities at the ventilation system inlets: u_1_ = 0.39 m/s and u_2_ = 0.15 m/s.

To investigate the effect of airflow rate on particle distribution, the initial inlet velocities, $u_{1}=1.69\;m/s$ and $u_{2}=0.64\;m/s$, which corresponded to twenty-six air changes per hour, were reduced by 76%, reaching five air changes per hour. This adjustment allowed for a more detailed analysis of how lower airflow rates affect the dispersion and accumulation of ultrafine particles in the indoor environment. The inlet conditions for the ventilation system air ducts were set at velocities of $u_{1}=0.39\;m/s$ and $u_{2}=0.15\;m/s$, respectively, with a constant inlet temperature of 20 °C. From Figure 5, where the flow lines in the space are presented, the flow velocities range from $2.23\times 10^{-8}\;m/s$ to 0.842 m/s, with the highest values being located near the ventilation inlet and outlet areas. The reduction in inlet velocities, which was implemented to achieve a lower air exchange rate, led to an overall reduction in the flow velocities in the space.

**Figure 6:**
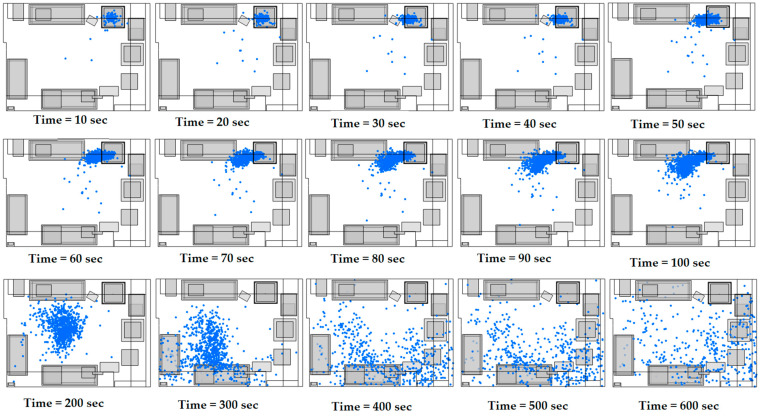
A top-down view of particle distribution in the 3D printing facility between 10 and 600 s, with air velocities at the ventilation system inlets: u_1_ = 0.39 m/s and u_2_ = 0.15 m/s.

In Figure 6, the distribution of ultrafine particles in the plan view of the space is observed, with the air velocities at the inlets of the ventilation system being u_1_ = 1.69 m/s and u_2_ = 0.64 m/s. In the initial stages (0–60 s), a sharp increase in the total number of particles is observed, which occurred due to the intense emission from the source. During this phase, the particles are mainly concentrated around the source, with minimal dispersion in the rest of the space. From 60 to 100 s, the particles continue to be mainly concentrated near the source, while their dispersion towards the rest of the space is limited. However, from 200 s onwards, their gradual removal from the source is observed, as they are directed towards the outlets of the ventilation system. At the final time points (300, 400, 500, and 600 s), the particles are mainly concentrated near the ventilation system outlets, with their overall concentration gradually decreasing. This development highlights the importance of ventilation mechanisms and airflow in the effective removal of particles. It is observed that in this case, where the velocities at the ventilation system inlets are lower, more time is required for the complete removal of particles from the space, which highlights the need for an adequate ventilation system design.

### 3.3. Simulations with Cooling Fans on the Print Head

The use of print head cooling fans is a critical operational parameter for evaluating system sensitivity and its effect on particle distribution [24]. Cooling fans in 3D printing play a critical role in improving print quality by promoting controlled cooling, which helps solidify the extruded filament and improves layer adhesion. Although not designed to remove particles, the airflow created by fans can indirectly affect the dispersion of particles within the print area. The speed and orientation of the fans can displace and disperse particles, creating a temporary increase in airflow velocity. To evaluate the applicability of our model in a variety of scenarios, we adapted the operation of two specific cooling fans, which correspond to the fans mounted on the 3D printer print heads. These fans have dimensions of 25 × 25 × 10 mm and are characterized by a rotation speed of 10,000 rpm and an airflow of 2.02 ft^3^/min (0.06 m^3^/min) (Table S1, Supplementary Information).

**Figure 7:**
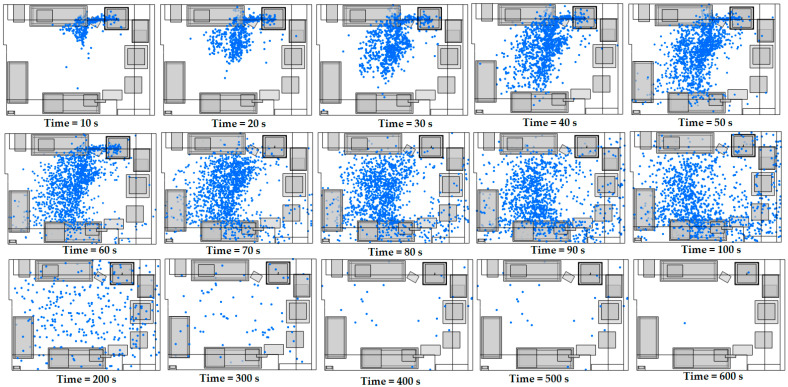
A top-down visualization of the particle distribution in the 3D printing room facility (with cooling fans on the print head) between 10 and 600 s with air velocities at the ventilation system inlets: u_1_ = 0.39 m/s and u_2_ = 0.15 m/s.

Figure 7 shows the distribution of ultrafine particles in the top view of the room, with the cooling fans mounted on the print head. In the initial phase, from 0 to 60 s, a sharp increase in the total number of particles is observed, as this period is associated with the active emission of particles from the source. During this time, the particles are mainly concentrated around the emission source, but already from the first few seconds, they start to disperse in the rest of the room, covering a large part of the space, due to the existence of the cooling fans. After the first 60 s and up to 300 s, the particles disperse uniformly and cease to remain concentrated near the emission source, indicating that their emission has ceased. At this stage, the distribution of particles expands and covers a larger area of the room, directed towards the outlets of the ventilation system. At the extraction points, increased levels of turbulent kinetic energy are observed, which facilitate dispersion and efficient air circulation, allowing particles to be removed from the room (Figure S5, Supplementary Information). At the final time points (400, 500, and 600 s), the particle concentration decreased significantly, leaving very few particles in the room. This observation shows that the ventilation mechanisms and the direction of the airflow contribute significantly to the removal of particles, reducing their concentration even in places where the flow is gentler.

**Figure 8:**
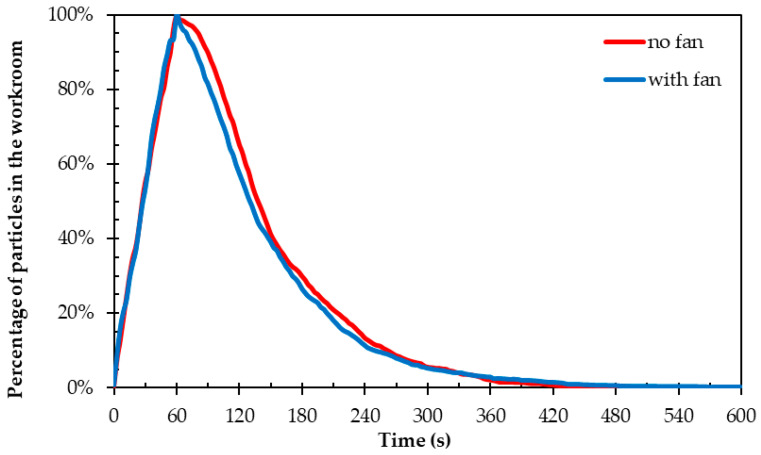
Proportion of particles in the workroom air during simulations (0–600 s), comparing scenarios with and without the cooling fan on the print head.

Figure 8 shows the comparison of particle concentration with and without fan operation versus time, expressed as a percentage of the maximum concentration in space. A sharp increase in concentration is observed in both cases, peaking at 60 s and indicating particle release. After the peak, the concentration begins to decrease, following a similar course for both curves, with the concentration in the fan case decreasing slightly faster. From 60 to 120 s, the presence of the fan contributes and creates a difference of up to 8% between the predicted particle concentrations. However, as the particles move away, the effect of the fan weakens, and both curves converge to values close to zero after about 420 s. This shows that although the fan speeds up the initial removal process of the particles, it does not significantly reduce the total time required to completely remove them from the space. In summary, the use of the fan offers a small improvement in particle removal speed in the initial stages but does not have a significant impact on overall cleaning time.

### 3.4. CFD Model Limitations

The accurate simulation of turbulent airflows in ventilated spaces is a significant challenge due to the existence of a wide range of eddy sizes and time scales. From large eddies, which are comparable to the dimensions of the space, to small-scale Kolmogorov eddies, turbulent flows are characterized by a hierarchy of scales that require the careful selection of computational methods to achieve reliable results [45].

The Reynolds-averaged Navier–Stokes method is based on the decomposition of the flow variables into average and fluctuating components, allowing for the use of models that simplify complex interactions in the flow. This approach is computationally economical and is a reliable method for many applications. However, the selection of the appropriate turbulent flow model remains a challenge, as it decisively affects the accuracy of the results. In contrast, the direct numerical simulation (DNS) method solves the Navier–Stokes equations numerically without the use of turbulence models. This means that all scales of turbulent flow, from the smallest (Kolmogorov scales) to the largest, are fully resolved on the computational grid. This approach offers excellent accuracy, but the computational cost is extremely high. Even at low Reynolds numbers, the computational resource requirements are enormous, making this method inapplicable for the high Reynolds numbers encountered in indoor ventilation spaces [50]. The large eddy simulation (LES) method offers a practical compromise between the accuracy of DNS and the computational efficiency of RANS. In this approach, large-scale turbulent motions are solved directly, while smaller scales are modeled using subgrid-scale (SGS) submodels. LES focuses on the detailed resolution of the most dynamically important structures, reducing the computational requirements compared to DNS. However, it remains computationally demanding, especially for applications such as indoor ventilation, where a high-resolution mesh is required near the walls, inlets, and outlets. Although LES is less accurate than DNS due to its reliance on modeling small-scale phenomena, it is considered a powerful research tool and is predicted to gain even greater application in the future, especially for flows involving separations and secondary flows, phenomena typical in indoor airflows [51].

The positioning of air ducts and ventilation openings, such as inlets and outlets, plays a pivotal role in directing airflow and determining its efficiency, which in turn impacts the distribution of particles throughout the space [52,53,54]. The room’s geometry also significantly affects airflow patterns, as factors like the room layout, ceiling height, and the presence of obstacles can induce turbulence or create stagnant zones, leading to an uneven particle distribution. Building structures often have complex geometries, such as irregular spaces, multiple rooms, and openings, as well as bulky objects, all of which significantly affect airflow. This complexity makes the natural flow of air more intricate, with airflows dispersed across rooms in ways that are not always straight or symmetrical. These factors further complicate the mechanism of turbulent diffusion. To accurately capture these dynamics and details, dense numerical grids and increased computational time are necessary. Moreover, the choice of an appropriate method for modeling particle transport is crucial for understanding and predicting airflow and particle dispersion accurately. Furthermore, the choice of the appropriate method for modeling particle transport plays a crucial role. The two widely used methods for simulating particle transport are the Lagrangian and Eulerian methods [51]. The Lagrangian method treats the particles as a discrete phase and calculates the trajectory of each particle, while the Eulerian method treats the particles as a continuous phase and approximates their behavior by solving the transport equations for a scalar concentration field. According to previous studies, the two methods provided similar results for particle scattering, but their computational costs were quite different [55].

Airflow velocity is a critical parameter influencing the redirection and dispersion of particles within an indoor environment [53,54]. Additionally, air stability, whether thermal instability or the presence of air currents, can influence particle concentration and diffusion, as unstable airflow may result in localized areas with a higher particle concentration. Specifically, in the context of 3D printing environments, as examined in this study, MacCuspie et al. [24] emphasize the need for further research to assess the sensitivity and impact of various printing process parameters on UFP dynamics. These parameters include print head motion, print head cooling fans, the movement of individuals within the space, heat sources, the simultaneous operation of multiple printers, and local ventilation. Building on these insights, our study focuses on investigating airflow speed by adjusting different inlet velocities in the air ventilation system, while also exploring two key printing process parameters: heat sources (such as the nozzle and print bed) and print head cooling fans. Heat sources generate convection currents due to the high temperatures required for melting filaments and heating the printing surface, while cooling fans create localized airflows that, depending on their orientation, power, and speed, can either enhance or limit the dispersion of UFPs.

CFD is a valuable tool for indoor flow analysis, as the collection of reliable experimental data requires careful experimental design, accurate measurements, and the ability to control variables that may affect the results. Experimental setups can be expensive and time-consuming, especially when simulating realistic scenarios such as indoor airflow. Measurements must be accurate and cover a variety of parameters such as air velocity, pressure, and temperature, which often require special equipment and techniques that can be difficult to apply. Furthermore, the interaction of flows and the presence of non-linear effects can complicate the experimental process, making it difficult to draw clear and useful conclusions [56,57]. Based on the above description of the challenges one faces when creating a computational model for the study of indoor airflow, we can understand that the accuracy of the results is significantly affected by the following parameters: the complexity of the geometry of the indoor spaces, the difficulty in modeling turbulent flows, choosing the method to model particle transport, and experimentally confirming the computational results.

In summary, building a reliable computational model for studying indoor airflow requires a careful balance between accuracy and computational cost. The choice of method and its implementation depend on the specific requirements of the application and the available computational resources. Model testing and refinement, combined with a case-by-case analysis, are necessary to ensure the accuracy of the results. In conclusion, this study examines the role of various factors, such as airflow velocity and 3D printing process parameters (cooling fans, printing temperatures), which affect the dispersion of ultrafine particles in the indoor environment. Future research should aim to extend these results by investigating a wider range of room configurations, ventilation strategies, and printing process parameters to improve our understanding of the behavior of UFP.

## 4. Conclusions and Future Potential

In conclusion, the CFD model developed to simulate airflow, particle dispersion, and heat transfer in a 3D printing room serves as a valuable tool for accurately representing the system’s dynamics. The model effectively depicts the expected airflow patterns, with lower velocities in the room’s center (average 0.14 m/s) and higher velocities near the ventilation inlets and outlets (average 2.065 m/s), accompanied by increased turbulent kinetic energy in these areas. These results align with typical airflow distribution patterns in enclosed spaces. Additionally, the simulation demonstrates that reducing inlet velocities by 76%, from twenty-six to five air changes per hour, compromises the ventilation system’s ability to maintain air quality. This reduction negatively impacts the system’s performance regarding particle diffusion, highlighting that inlet velocities and total airflow are critical factors for optimal particle distribution within the space. In addition, the simulation effectively captures the influence of cooling fans located on the 3D printer print head, where particle diffusion is greatly enhanced compared to cases without cooling fan activity, representing real particle behavior and particle behavior influenced by local sources of airflow. The presence of the fans accelerates the removal of particles in the initial 60–120 s, creating a difference of up to 8% in the predicted particle concentrations compared to the case without fans. However, as the particles move away from the influence of the fans, their contribution decreases. This suggests that although fans facilitate the initial removal of particles, they do not significantly affect the total time required for their complete removal from the space. Regarding thermal effects, the results show that the heat generated by the 3D printer causes minimal overall variation in the room temperature. A small local temperature increase is observed, reaching approximately 40 °C, mainly at the level of the print bed and in the areas around the print head. This behavior is in line with expectations for the thermal performance of the printer, given the scale of the heat generated. Heat sources affect particle dispersion mainly during the first 60–120 s after their emission, where a slightly increased dispersion is observed. However, as the cleaning process by the ventilation system progresses, the effect of the heat sources weakens and stops affecting the distribution and dispersion of the particles. Nevertheless, the model shows that even this limited local heating can affect the particle diffusion patterns. This finding highlights the importance of temperature gradients and thermal currents in particle dispersion, which may have practical implications for the design of ventilation systems and indoor air quality management.

In our study, we do not have experimental data to validate our results, making the evaluation of particle transport and ventilation systems more complicated. While our model provides a reliable estimate of airflow and particle distribution for the specified parameters, as the flow lines and particle distribution envelopes are consistent with those in the literature [58,59,60,61], it cannot be generalized to all 3D printing environments without further validation from experimental data. Despite these limitations, our model provides a realistic profile of airflow and particle dispersion in the study space and serves as a valuable foundation for future studies on ventilation design and particle diffusion.

Future studies should focus on key parameters to better understand their influence on particle behavior in 3D printing environments, which is critical for worker safety. Some of the critical factors that require investigation are (1) the effect of print head movement, (2) the presence of moving people, (3) the operation of multiple printers simultaneously, and (4) the effect of enclosures, which act as physical barriers and significantly reduce the dispersion of particles into the surrounding air, (5) different printing temperatures, (6) as well as multiple print models with different cooling systems. Comparing these findings with experimental data is essential to validate computational models to ensure their accuracy in real-world conditions. As future research identifies the factors with the greatest impact on exposure, this knowledge will help improve worker safety by reducing their exposure to hazardous particle concentrations and improving working conditions in modern industrial environments.

## Figures and Tables

**Table 1:** Mesh properties for coarse, medium, and fine resolutions.

	Coarse	Medium	Fine
Number of elements	647,759	1,654,901	5,584,269
Minimum element size	$2.06\times 10^{-3}\;\;(m)$	$9.81\times 10^{-4}\;\;(m)$	$9.78\times 10^{-4}\;\;(m)$
Maximum element size	0.461 (m)	0.366 (m)	0.285 (m)
Minimum element quality	0.0118	0.0177	0.0272
Average element quality	0.6408	0.6559	0.6718

**Table 2:** Calculation of the grid convergence index.

Number of Elements	Velocity	r	p	$e_{\alpha }$	GCI
$5,584,269\;(\;N_{1}$)	2.217 ($c_{1}$)	2.55 ($r_{21}$)	2	0.06 ($e_{\alpha }^{21}$)	8% (${GCI}_{21}$)
1,654,901 ($N_{2}$)	2.09 ($c_{2}$)	3.37 ($r_{32}$)		0.16 ($e_{\alpha }^{32}$)	24% (${GCI}_{32}$)
$64,775\;(N_{3}$)	1.75 ($c_{3}$)	-		-	-

## Data Availability

Data are contained within the article and Supplementary Materials.

## Supplementary Materials

The following supporting information can be downloaded at the following website: https://www.mdpi.com/article/10.3390/ma18020262/s1, Figure S1: Method for network measurements in a rectangular ventilation system inlet cross-section; Figure S2: Turbulent kinetic energy distribution (a) from the outlet side of the ventilation system in the room and (b) from the inlet side of the ventilation system in the room; Figure S3: Top-down visualization of the particle distribution in the 3D printing room setup (with and without heat sources) between 10 and 600 s; Figure S4: Particle percentage in the workplace air with (thermal) and without heat sources (No thermal), throughout the simulations (0–600 s); Figure S5: Turbulent kinetic energy distribution with cooling fans on the print head (a) from the outlet side of the ventilation system in the room and (b) from the inlet side of the ventilation system in the room; Table S1: Boundary conditions and parameters used in the simulations; Table S2: Experimental measurements of the velocities at the inlets of the ventilation system.
