# Supplementary material for: Computational Fluid Dynamics as a Digital Tool for Enhancing Safety Uptake in Advanced Manufacturing Environments Within a Safe-by-Design Strategy
Source: Materials (Basel). 2025 Jan 9;18(2):262. doi: 10.3390/ma18020262 (PMC11766716; doi:10.3390/ma18020262)
Supplement: Supplementary file 1 [file materials-18-00262-s001.zip › materials-3351657-supplementary.pdf]

# Supplementary Information

# Computational Fluid Dynamics Modeling for Enhancing Safety Uptake in Advanced Manufacturing Environments

Dionysia M. Voultsou, Stratos Saliakas, Spyridon Damilos and Elias P. Koumoulos*

*Innovation in Research & Engineering Solutions (IRES), 1000 Brussels, Belgium*
** Correspondence: epk@innovation-res.eu*

## Contents

1 Boundary Conditions ........ 2

1.1 Boundary conditions and parameters for the two steps of the FEM model. 2

1.2 Experimental measurements of intake port velocities of the ventilation system 4

2 Airflow, heat and particle dispersion simulations........ 6

3 Simulations with cooling fans on the printhead ........ 8

4 References ........ 9

# 1 Boundary Conditions

## 1.1 Boundary conditions and parameters for the two steps of the FEM model

**Table S1** details the boundary conditions parameters applied to the computational model to simulate airflow, thermal behavior of the system, as well as particle detection.

**Table S1.** Boundary conditions and parameters used in the simulations.

| **Non-isothermal flow study** | |
|---|---|
| **Boundary condition** | **Value / Description** |
| Inlet | Intake port (1) : $u_1 = 1.69\ m/s$<br>Intake port (2) : $u_2 = 0.64\ m/s$<br><br>Experimental measurements (**Table S2**) |
| Outlet | Atmospheric pressure, $p_{o1} = 0\ Pa$ with backflow suppression enabled to prevent any flow reversal |
| Wall Conditions | No-slip condition applied on all walls $u = 0$ |
| Thermal Boundary Conditions | Printing bed temperature: $T_{bed} = 80°\text{C}$ [1]<br>Nozzle temperature: $T_{nozzle} = 250°\text{C}$ |
| Ambient Temperature | Maintained at $\text{T}_{\text{ambient}} = 20°\text{C}$ to simulate thermal exchange with the environment |
| Wall Conditions | Adiabatic walls $(\boldsymbol{q} = 0)$ |
| Cooling fans | 2.02 ft³/min (0.06 m³/min) (*Model Number D2510M05BPLB1b-5, 2510-5 Series 0.95 Watt (W) Power Brushless Direct Current (DC) Axial Fan Pelomis techonologies.INC*) |
| **Particle Tracing for fluid flow study** | |
| **Boundary condition** | **Value / Description** |
| Inlet | Release Times [Start: 0 s, Step: 0,1 s, Stop: 60 s]<br><br>Number of particles per release N=10 |
| Outlet | Set to "Disappear"<br><br>For Disappear it means that the particles are not visualized once they strike the outlet boundary. |
| Wall Conditions | Diffuse Scattering condition to simulate realistic particle-wall interactions<br><br>$V_{t,1} = \lvert \boldsymbol{V}_C \rvert \sin\theta \cos\varphi$<br><br>$V_{t,2} = \lvert \boldsymbol{V}_C \rvert \sin\theta \cos\varphi$<br><br>$V_n = \lvert \boldsymbol{V}_C \rvert \cos\theta$<br><br>$\varphi\epsilon[0,2\pi]$, $\theta\epsilon\left[0,\frac{\pi}{2}\right]$,$f(\theta,\varphi) = \frac{1}{\pi}\cos\theta\sin\theta$<br><br>Where $\boldsymbol{V}_C$is the particle velocity when striking the wall<br><br>θ is the angle between the direction of the reflected particle and the wall normal |
| Gravity Force | $\boldsymbol{g} = 9.80665\ m/s^2$ |
| Particle Properties | $\text{d}_\text{p} = 30\ \text{nm}$ [1]<br>$\rho_p = 1040\ kg/m^3$ (based on acrylonitrile butadiene styrene (ABS) material properties) |

## 1.2 Experimental measurements of intake port velocities of the ventilation system

The experimental method used to collect measurements on a rectangular cross-section is based on creating a grid with rectangles of equal area. The cross-section of the rectangular duct is divided into these equal sections, with the measurement points placed on the centroidal axis of each section. The objective of the method is to record velocities at representative points, which are uniformly distributed throughout the pipeline cross-section, thus providing an accurate and representative depiction of the velocity profile in the pipeline. **Figure S1** shows the configuration of a measurement grid for the collection of experimental data at a rectangular cross-section orifice of the ventilation system.

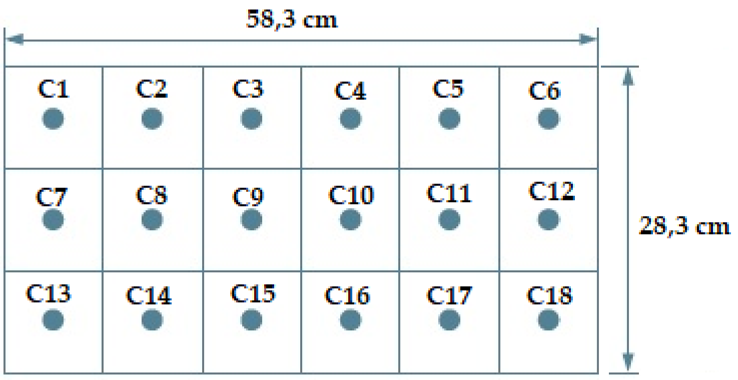


**Figure S1.** Method for network measurements in a rectangular ventilation system inlet cross-section.

**Table S2** shows the experimental measurements taken from the two rectangular inlets of the room ventilation system. The average value of these measurements corresponds to the velocity boundary conditions, which are used as inputs to the computational model to simulate the air flow inside the room caused by the ventilation system. The Kestrel 5400 instrument (*Kestrel Meters, Boothwyn, PA, USA*) was used for the experimental measurements.

`

**Table S2.** Experimental measurements of the velocities at the inlets of the ventilation system.

| **Intake port 1** | | **Intake port 2** | |
|---|---|---|---|
| **Measurement** | **Velocity (m/sec)** | **Measurement** | **Velocity (m/sec)** |
| C1 | 2.3 | C1 | 1.1 |
| C2 | 2.2 | C2 | 0.8 |
| C3 | 2.0 | C3 | 0.4 |
| C4 | 2.1 | C4 | 0.4 |
| C5 | 1.8 | C5 | 0 |
| C6 | 1.7 | C6 | 0 |
| C7 | 2.1 | C7 | 1.2 |
| C8 | 2.1 | C8 | 1.1 |
| C9 | 2.0 | C9 | 1.1 |
| C10 | 1.4 | C10 | 0.9 |
| C11 | 1.6 | C11 | 0.9 |
| C12 | 1.5 | C12 | 0.5 |
| C13 | 1.4 | C13 | 0.7 |
| C14 | 1.2 | C14 | 0.6 |
| C15 | 1.2 | C15 | 0.5 |
| C16 | 1.5 | C16 | 0.5 |
| C17 | 1.3 | C17 | 0.6 |
| C18 | 1.1 | C18 | 0.3 |

## 2 Airflow, heat and particle dispersion simulations

**Figure S2** shows the distribution of turbulent kinetic energy in slices inside the room and for the study case without cooling fans on the print head. Turbulent kinetic energy is a fundamental parameter in fluid flow analysis, particularly in turbulent (or unsteady) flows. Turbulent kinetic energy represents the energy contained in the turbulent eddies of a fluid and is directly related to the intensity of velocity disturbances. Simply put, it shows the "eddy" or turbulent nature of the flow, i.e. how intense and unsteady the fluid motion is at various points in the flow.

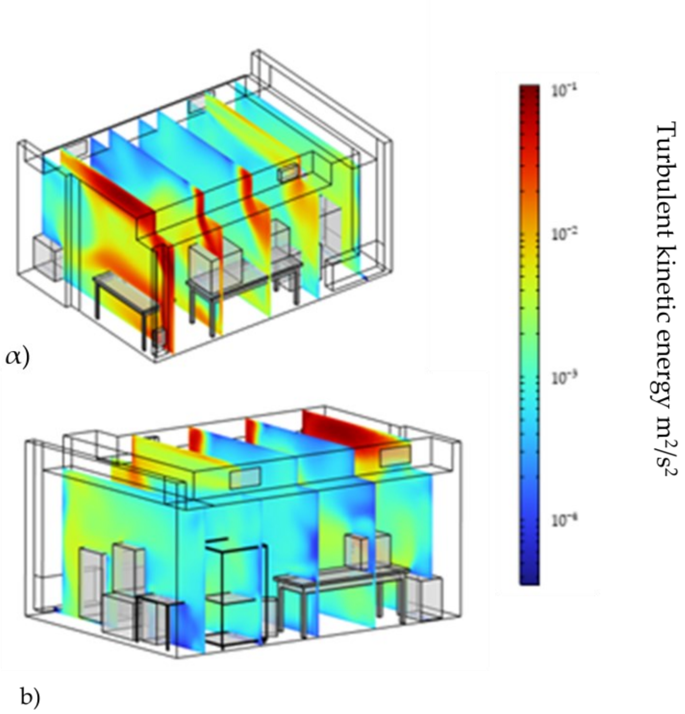


**Figure S2.** Turbulent kinetic energy distribution a) from the outlet side of the ventilation system in the room b) from the inlet side of the ventilation system in the room.

**Figure S3** and **Figure S4** illustrate the distribution of particles and their corresponding concentration in space, both with and without heat sources. Temperature plays a decisive role in the distribution of air within the interior, as well as in the dispersion of particles. The presence of heat directly affects air flow, causing upward motions that may enhance pollutant concentration. Therefore, temperature is one of the main factors that determine the distribution and concentration of particles, significantly affecting indoor air quality.

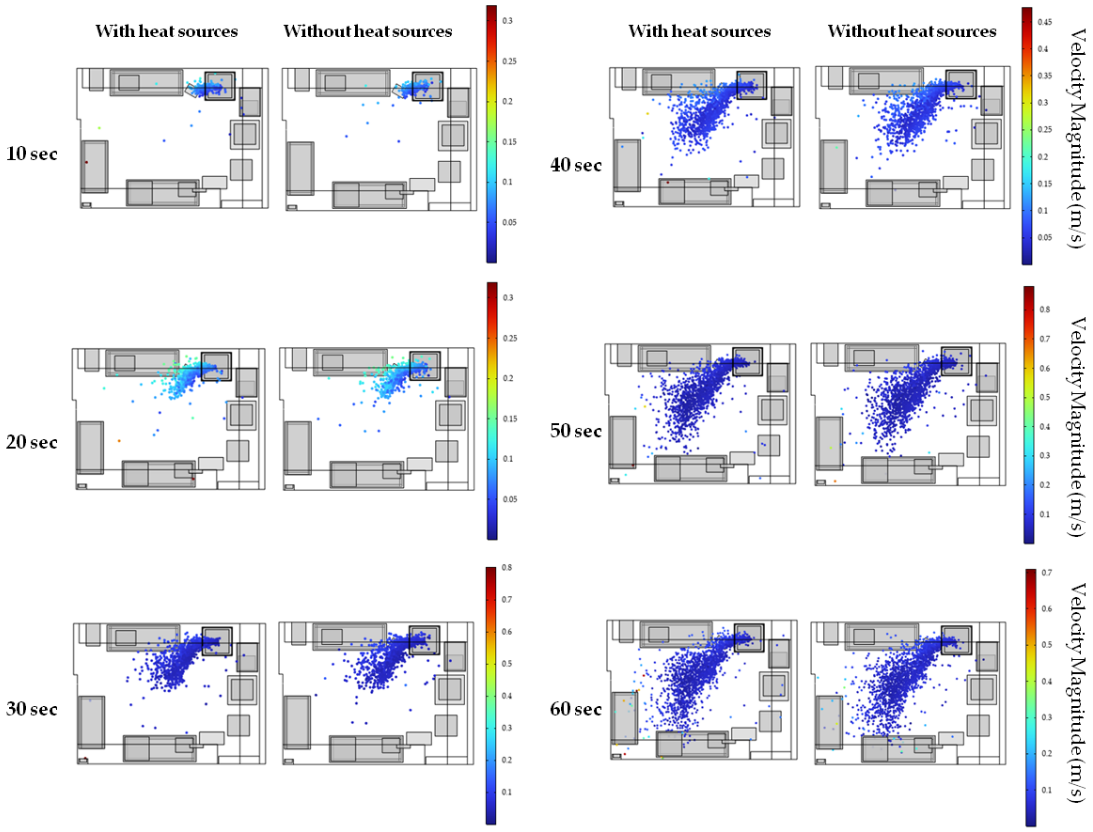


**Figure S3.** Top-down visualization of the particle distribution in the 3D printing room setup (with and without heat sources) between 10-600 s.

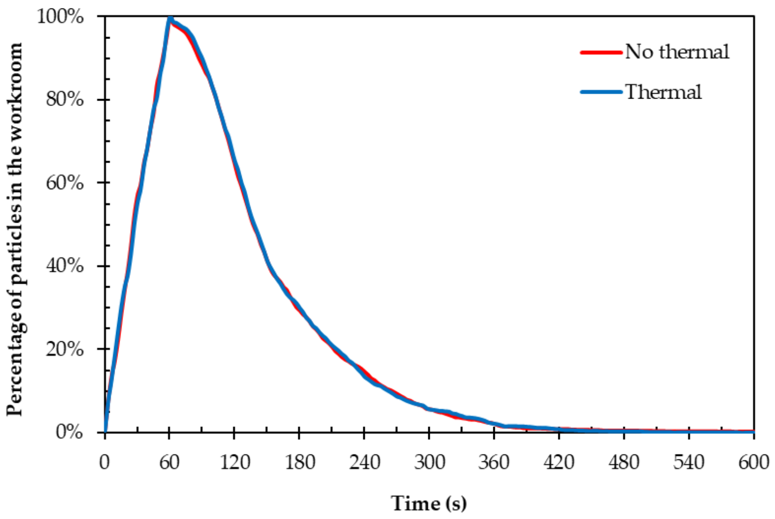


**Figure S4**. Particle percentage in the workplace air with (Thermal) and without heat sources (No thermal), throughout the simulations (0-600 s).

## 3 Simulations with cooling fans on the printhead

**Figure S5** shows the distribution of turbulent kinetic energy in slices inside the room and for the study case with cooling fans on the print head.

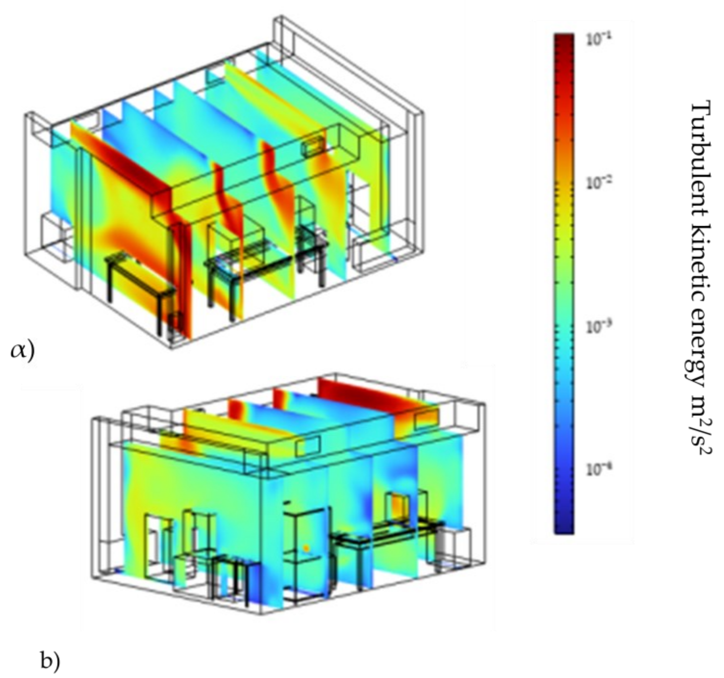


**Figure S5.** Turbulent kinetic energy distribution with cooling fans on the printhead a) from the outlet side of the ventilation system in the room b) from the inlet side of the ventilation system in the room.

## 4 References

1. Gu, J.; Wensing, M.; Uhde, E.; Salthammer, T. Characterization of Particulate and Gaseous Pollutants Emitted during Operation of a Desktop 3D Printer. *Environment International* **2019**, *123*, 476–485, doi:10.1016/j.envint.2018.12.014.
